# Supplementary figures and images for: X-ray structure and enzymatic study of a bacterial NADPH oxidase highlight the activation mechanism of eukaryotic NOX
Source: eLife. 2024 Apr 19;13:RP93759. doi: 10.7554/eLife.93759 (PMC11031084; doi:10.7554/eLife.93759)

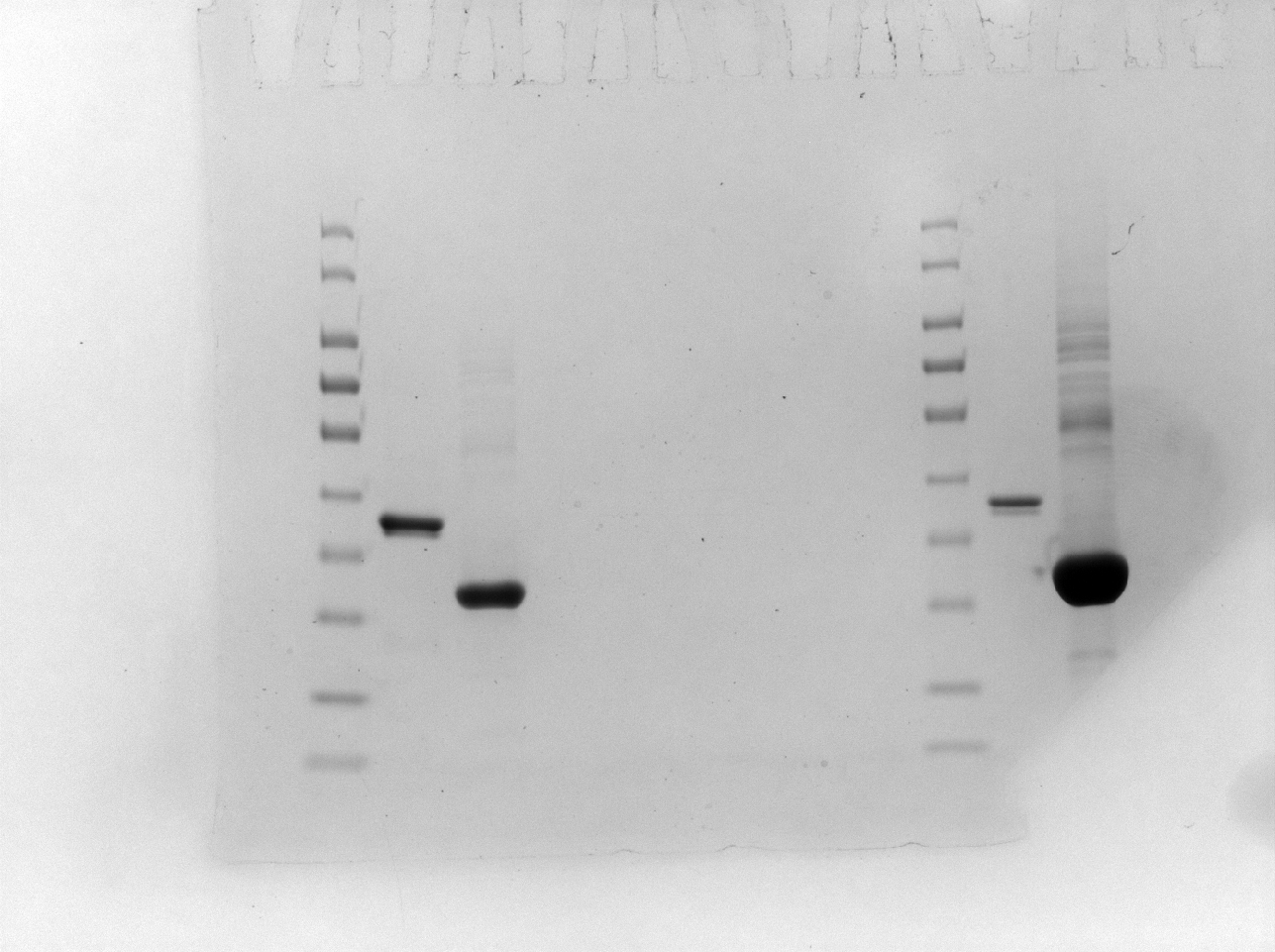

Supplement: Figure 1—figure supplement 1—source data 1. — Redundancy corresponds to a different quantity loaded on the gel. [file elife-93759-fig1-figsupp1-data1.tiff]
