# Supplementary material for: X-ray structure and enzymatic study of a bacterial NADPH oxidase highlight the activation mechanism of eukaryotic NOX
Source: eLife. 2024 Apr 19;13:RP93759. doi: 10.7554/eLife.93759 (PMC11031084; doi:10.7554/eLife.93759)
Supplement: Table 2—source data 1. [file elife-93759-table2-data1.pdf]

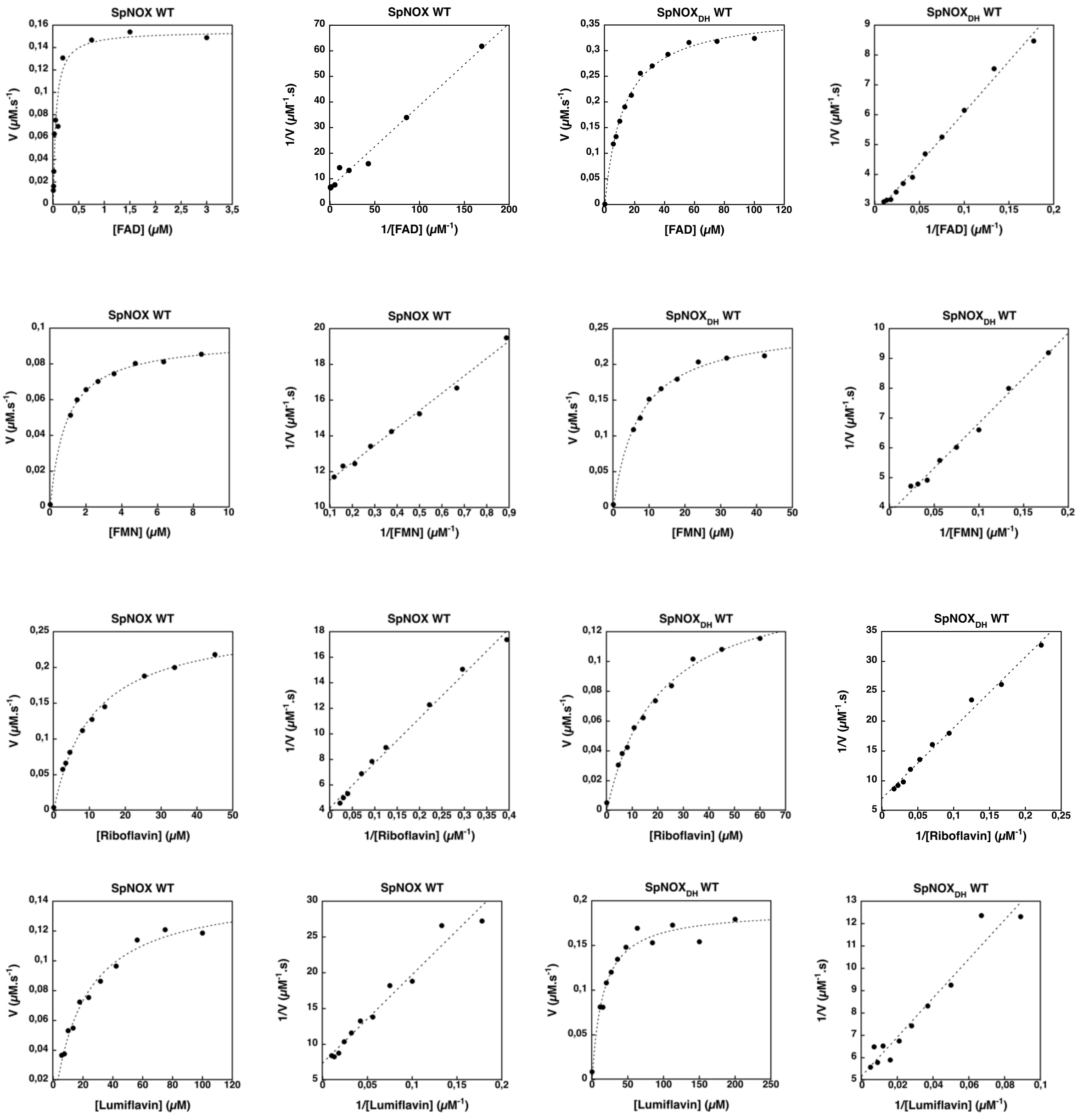

Table 2-source data 1. Michaelis Menten analysis of SpNOX and SpNOX$_{DH}$ as a function of the flavin substrate. Cytochrome *c* reductase activity was monitored at 550 nm. For all flavin substrates the Michaelis Menten plots and the Lineweaver-Burk plots are presented. K_m and k_{cat} extracted from these experiments are presented in Table 2.
