# Supplementary material for: X-ray structure and enzymatic study of a bacterial NADPH oxidase highlight the activation mechanism of eukaryotic NOX
Source: eLife. 2024 Apr 19;13:RP93759. doi: 10.7554/eLife.93759 (PMC11031084; doi:10.7554/eLife.93759)
Supplement: Table 3—source data 1. [file elife-93759-table3-data1.pdf]

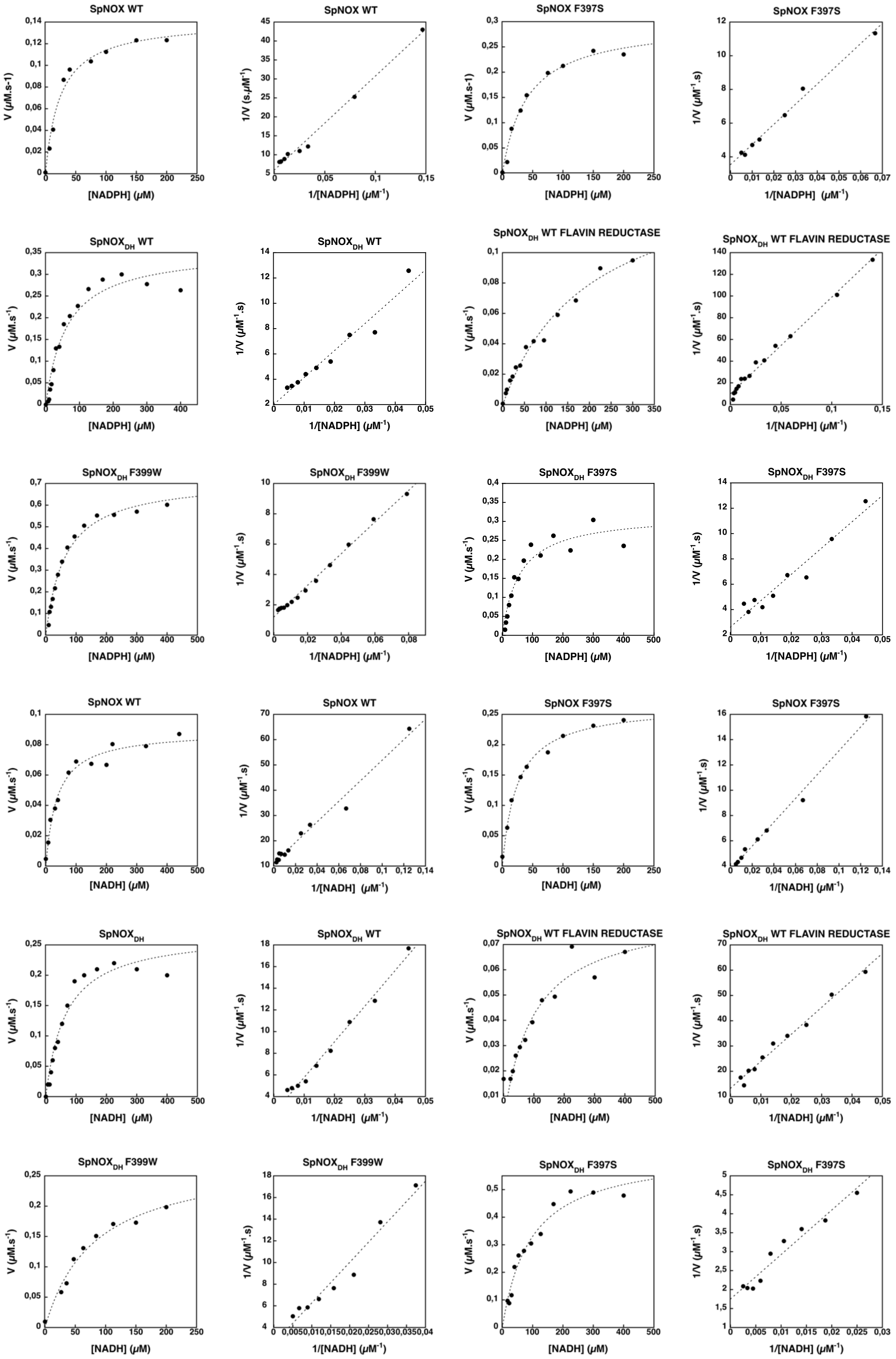

Table 3-source data 1. Michaelis Menten analysis of wild type and mutant SpNOX and $SpNOX_{DH}$ as a function of the nicotinamide-based electron donor. Cytochrome *c* reductase activity was monitored at 550 nm, and, in panels so labeled, flavin reductase activity was monitored at 340 nm. For all flavin substrates the Michaelis Menten plots and the Lineweaver-Burk plots are presented. K_m and k_{cat} extracted from these experiments are presented in Table 3.
