# Supplementary material for: X-ray structure and enzymatic study of a bacterial NADPH oxidase highlight the activation mechanism of eukaryotic NOX
Source: eLife. 2024 Apr 19;13:RP93759. doi: 10.7554/eLife.93759 (PMC11031084; doi:10.7554/eLife.93759)
Supplement: Table 4—source data 1. [file elife-93759-table4-data1.pdf]

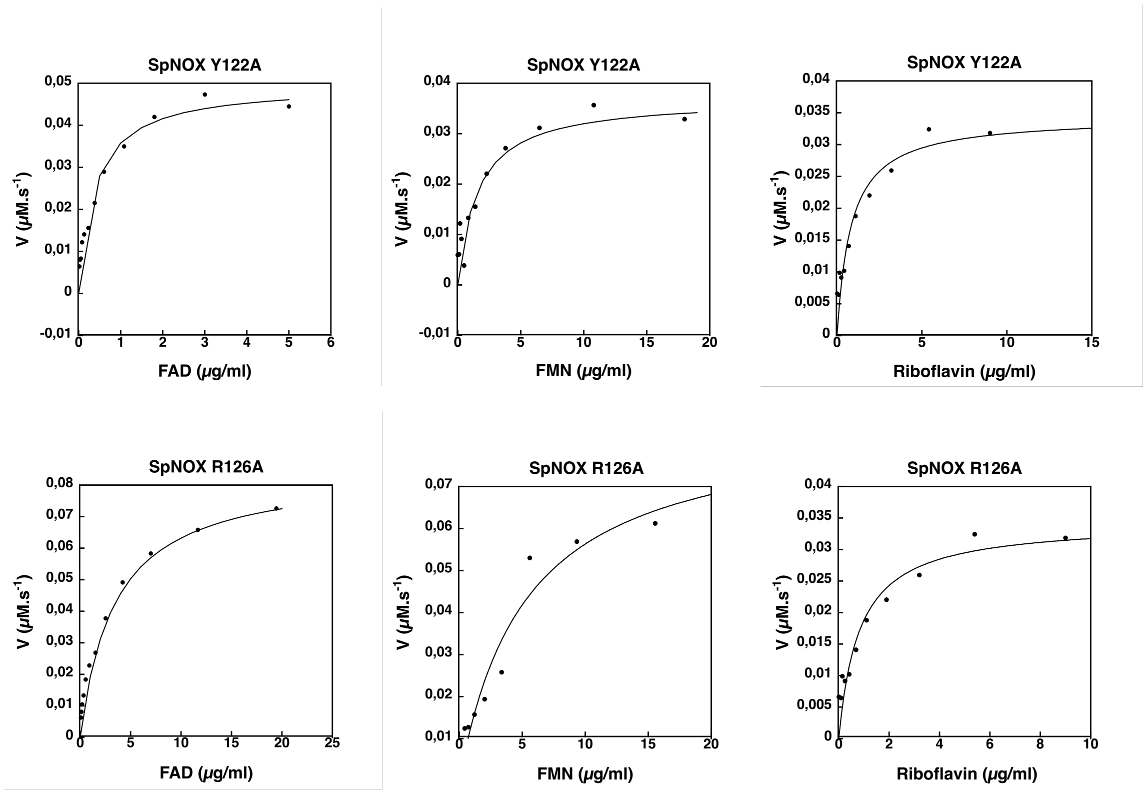

Table 4-source data 1. Michaelis Menten analysis of SpNOX and SpNOX Y122A and SpNOX R126A as a function of the flavin substrate. Cytochrome *c* reductase activity was monitored at 550 nm. For all substrates the Michaelis Menten plots are presented.
