# Supplementary material for: X-ray structure and enzymatic study of a bacterial NADPH oxidase highlight the activation mechanism of eukaryotic NOX
Source: eLife. 2024 Apr 19;13:RP93759. doi: 10.7554/eLife.93759 (PMC11031084; doi:10.7554/eLife.93759)
Supplement: Table 4—source data 2. [file elife-93759-table4-data2.pdf]

	k_{cat} (s-1)		
	**SpNOX WT**	**Y122A**	**R126A**
FAD	4,23 ± 0.3	1,92 ± 0.1	3,5 ± 0.1
FMN	2,46 ± 0.2	1,42 ± 0.1	2,5 ± 0.1
Riboflavin	3,23 ± 0.1	1,31 ± 0.1	1,85 ± 0.1

Table 4-source data 2. k_{cat} determined as a function of the mutation in the D-loop and the flavin used. These parameters were extracted from data of (Table 4-figure supplement 1).
